# A comparative study on efficacies of posterior microscopic mini-open and open technique for thoracolumbar burst fractures with severe traumatic spinal stenosis

**DOI:** 10.1186/s13018-022-03412-x

**Published:** 2022-12-01

**Authors:** Bin Zhang, Yanna Zhou, Hua Zou, Zimo Lu, Xin Wang, Jun Ao

**Affiliations:** 1 Department of Orthopaedics, Guizhou Province Osteological Hospital, 123 Shachong South Road, Guiyang, 550002 Guizhou Province People’s Republic of China; 2 Department of Orthopaedic Surgery, Affiliated Hospital of Zunyi Medical University, Zunyi, 563000 Guizhou Province People’s Republic of China; 3 Department of Epidemiology and Statistics, School of Public Health, Zunyi Medical University, Zunyi, 563000 Guizhou Province People’s Republic of China; 4 Department of Orthopaedics, Guizhou Aerospace Hospital, Zunyi, 563000 Guizhou Province People’s Republic of China

**Keywords:** Thoracolumbar fracture, Spinal stenosis, Surgical microscope, Minimally invasive

## Abstract

**Purpose:**

This study compares the efficacies of minimally invasive decompression by posterior microscopic mini-open technique combined with percutaneous pedicle fixation (hereafter MOT) to traditional open surgery in patients with severe traumatic spinal canal stenosis resulting from Arbeitsgemeinschaft für Osteosynthesefragen (AO) type A3 or A4 thoracolumbar burst fractures and provides references for clinical treatment.

**Methods:**

In total, 133 patients with severe traumatic spinal canal stenosis caused by AO type A3 or A4 thoracolumbar burst fractures who underwent MOT (group A) or traditional open surgery (group B) were retrospectively enrolled. The demographic and radiological data of the two groups were analyzed and compared.

**Results:**

A total of 64 patients were finally recruited in this study. There were no significant differences in gender, age, follow-up time, injury mechanism, injury level, AO classification, American Spinal Injury Association (ASIA) score, visual analogue scale (VAS) score, and duration of hospital stay between the two groups (*P* > 0.05). After the procedures, the prevertebral height ratio (PHR), the Cobb angle, and the mid-sagittal canal diameter compression ratio (MSDCR) were significantly improved (*P* < 0.05) in both groups. However, group A demonstrated less intraoperative bleeding and a greater VAS score improvement postoperatively and at the last follow-up but involved a longer operation time (*P* < 0.05). The PHR and the Cobb angle in the two groups showed no significant difference postoperatively and at the last follow-up (*P* > 0.05). In contrast, a significant improvement in MSDCR was observed at the last follow-up when compared with the postoperative value (*P* < 0.05). However, the Cobb angle in group A was better maintained than in group B at the last follow-up (*P* < 0.05), while the MSDCR in group B demonstrated a greater improvement at the last follow-up than in group A (*P* < 0.05).

**Conclusions:**

Both the MOT and traditional open surgery are effective treatment options for AO type A3 and A4 thoracolumbar burst fractures with severe traumatic spinal stenosis. The advantages of MOT include the minimally invasive procedure, extremely fine spinal canal decompression, less intraoperative bleeding, and significant pain relief. We suggest that MOT should be preferentially performed for AO type A3 or A4 thoracolumbar burst fractures with severe traumatic spinal stenosis.

## Introduction

90% of all spinal fractures occur in the thoracolumbar region, and burst fractures account for approximately 60% of cases [1]. Thoracolumbar fractures can cause serious spinal cord injury [2]. Decompression surgery alleviates secondary spinal cord injury and improves neurological recovery after acute spinal cord injury. Short-segment posterior fixation, especially percutaneous minimally invasive fixation, is well accepted for thoracolumbar fractures [3–6]. However, decompression and bone grafting are challenging in patients with a mid-sagittal canal diameter compression ratio (MSDCR) exceeding 50% [3, 7].

To date, the efficacies of minimally invasive decompression by posterior microscopic mini-open technique combined with percutaneous pedicle fixation (hereafter MOT) and traditional open surgery for thoracolumbar burst fractures with severe traumatic spinal canal stenosis have rarely been reported. The current study compares the efficacies of the two surgical modalities.

## Methods

### Demographics

From January 2012 to January 2018, 133 consecutive patients with severe traumatic spinal canal stenosis caused by Arbeitsgemeinschaft für Osteosynthesefragen (AO) type A3 or A4 thoracolumbar burst fractures underwent MOT (group A) or traditional open surgeries (group B). Sixty-four patients completed the 1-year follow-ups and were retrospectively enrolled in this study. The inclusion criteria were: (I) patients with single segmental AO type A3 or A4 thoracolumbar fractures; (II) intra-canal fracture fragments causing canal compromise, MSDCR > 50%; (III) no severe damage to the adjacent disks; (IV) the posterior ligamentous complex (PLC) was not severely damaged; and (V) the patients were admitted within 2 weeks of injury. The exclusion criteria were: (I) patients with significant osteoporosis, endocrine diseases, tuberculosis, or other diseases which may affect the vertebral structure; (II) patients with congenital spinal stenosis; (III) patients with incomplete clinical records; and (IV) a follow-up period shorter than 12 months.

### Clinical and radiographic records

All patient clinical and radiographic data were recorded at admission, postoperatively, and at the last follow-up (12–24 months after surgery). The clinical records included the general date, follow-up time, injury mechanism, hospital stay, operation time, intraoperative bleeding volume, visual analogue scale (VAS) score [8], American Spinal Injury Association (ASIA) scores [9], and AO spine injury classification [10].

**Fig. 1:**
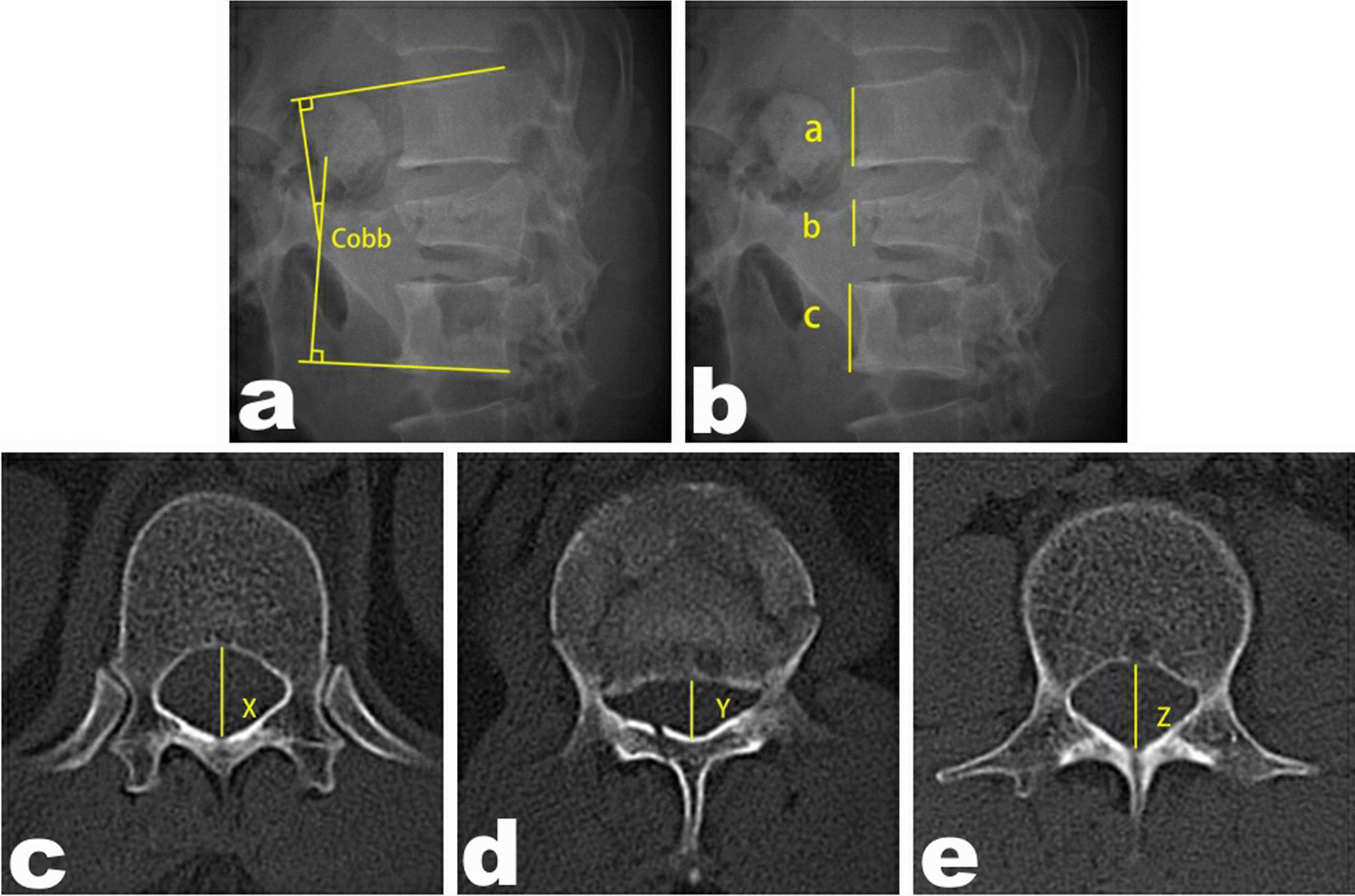
Imaging evaluation method. **a** Cobb angle. **b** PHR = 2b/(a + c)100%. **c–e** MSDCR = [1-2Y/(X + Z)]100%; *PHR* prevertebral height ratio, *MSDCR* mid-sagittal canal diameter compression ratio

Radiographic data included the prevertebral height ratio (PHR) [3], the injured vertebral Cobb angle, and the mid-sagittal canal diameter compression ratio (MSDCR) [11]. See Fig. 1.

### MOT methods

**Fig. 2:**
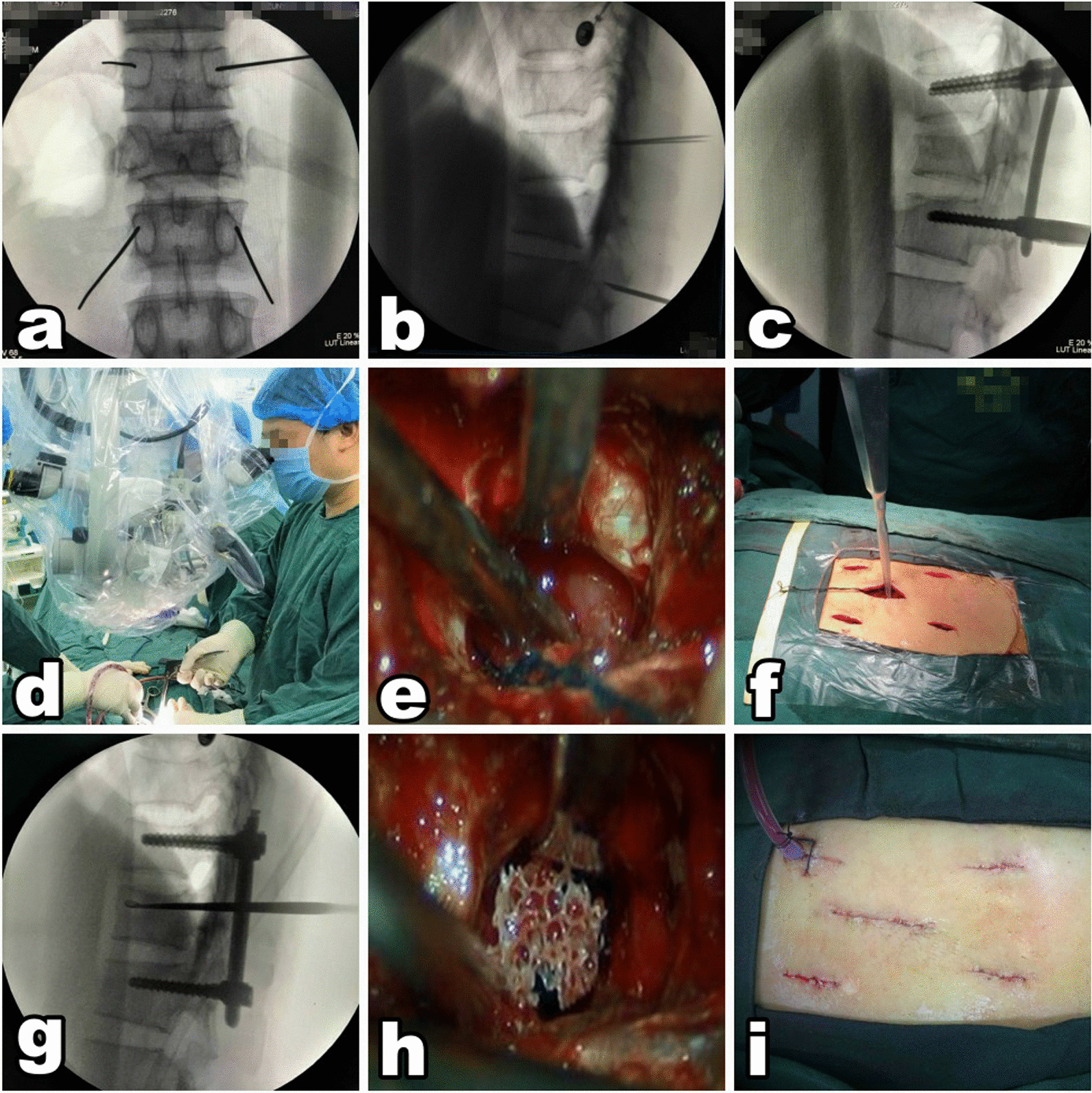
MOT methods **a, b** four guide needles were inserted into the adjacent vertebral pedicles under fluoroscopic guidance; **c** the positions of the percutaneous pedicle screws were confirmed radiographically after implantation; **d** fenestration of the vertebral lamina was performed via the 3 cm incision under microscopy; **e** visualizing and repositioning the fracture fragments into the vertebral body under microscopy; **f** using a curette to pry the injured endplates to achieve anterior and middle column reduction; **g** the satisfactory reduction was confirmed by lateral radiograph; **h** autologous bones and allograft bones were implanted in the injured vertebral body under microscopy; **i** a drainage tube was placed, and the incision was intradermally sutured

After general anesthesia, the patient was placed in a prone position, and the abdomen was suspended. Percutaneous pedicle screw fixation was performed under fluoroscopic guidance; percutaneous pedicle screws were placed in the superior and inferior vertebra adjacent to the injured vertebra. An approximately 3 cm posterior midline incision, centered on the injured vertebra, was performed to expose the lamina space of the injured vertebra. Fenestration of the vertebral lamina was then completed under microscopy, and the fracture fragments were visualized and repositioned into the vertebral body using the L-shaped operative tool, followed by longitudinal distraction of the vertebral body anterior margin. Subsequently, a curette was used to identify the fracture lines at the posterior wall of the injured vertebral body. Anterior and middle column reduction was performed and a 5-mm-diameter bone grafting channel was prepared. Proper reduction was confirmed using fluoroscopy, and autologous bones or allograft bones were implanted in the injured vertebral body via the bone grafting channel 3–4 mm deep from the posterior wall of the injured vertebral body. After adequate hemostasis, the wound was flushed, and a drainage tube was placed as necessary. The incisions were sutured layer by layer. See Fig. 2.

### Statistical analysis

The SPSS20.0 statistical software (IBM Corp., Armonk, NY, USA) was used for data analysis. Quantitative data were presented as mean ± standard deviation. Within-group comparisons of PHR, Cobb angle, MSDCR, and VAS score at multiple time points were analyzed with repeated measures analysis of variance combined with Bonferroni correction. Mann–Whitney U test was used for ASIA scores within-group comparisons. Age, follow-up time, hospital stay, operation time, intraoperative bleeding volume, PHR, Cobb angle, MSDCR, and VAS score between the two groups were compared by independent-sample t tests. Gender, injury mechanism, injury level, and AO classification between the two groups were compared by chi-square test. A probability less than 0.05 was considered statistically significant.

## Results

### Comparison of general conditions

A total of 64 patients were finally recruited; 69 patients were excluded due to incomplete clinical records or lost to follow-up. These 64 patients included 28 patients who underwent MOT and were classified as group A (21 males and 7 females), and 36 patients who underwent traditional open surgeries and were classified as group B (25 males and 11 females). Statistical analysis revealed no significant intergroup differences in gender, age, follow-up time, injury mechanism, injured level, AO classification, ASIA score, and MSDCR between the two groups (all *P* > 0.05, Table 1).

**Table 1:** Comparison of general conditions

Variables	Group A (n = 28)	Group B (n = 36)	Statistical value	*P* value
*Gender*				
Male	21	25	*χ*^2^ = 0.240	0.624
Female	7	11		
Age (years)	42.11 ± 10.77	41.64 ± 13.23	*t* = 0.152	0.880
Follow-up time (days)	16.46 ± 4.01	15.72 ± 4.91	*t* = 0.648	0.519
*Injury mechanism*				
Fall from height	21	26	*χ*^2^ = 1.345	0.789
Car accident	4	6		
Fall	2	1		
Heavy object smashing injury	1	3		
*Injured level*				
T11	1	2	*χ*^2^ = 1.529	0.738
T12	4	6		
L1	14	21		
L2	9	7		
*AO classification*				
A3	19	26	*χ*^2^ = 0.144	0.705
A4	9	10		
*ASIA score*				
A	0	8	*Z* = 1.570	0.116
B	0	6		
C	12	5		
D	13	11		
E	3	6		
MSDCR (%)	55.91 ± 6.70	56.21 ± 7.10	*t* = 0.167	0.868

*ASIA* American Spinal Injury Association, *AO* Arbeitsgemeinschaft für Osteosynthesefragen, *MSDCR* mid-sagittal canal diameter compression ratio, *Group A* MOT group, *Group B* traditional open surgery group

### Perioperative data

All procedures were completed successfully. No significant difference was found concerning the duration of hospital stay between both groups (*P* > 0.05, *Table **2*). A longer mean operative time and lower mean intraoperative bleeding volume were found in group A compared to group B. The differences were statistically significant (all *P* < 0.05).

**Table 2:** Comparison of hospital stay, operation time, intraoperative bleeding volume

Time	Group A (n = 28)	Group B (n = 36)	*t* values	*P* values
Hospital stay (days)	12.54 ± 3.04	13.89 ± 3.76	1.550	0.126
Operation time (min)	216.39 ± 38.11	165.22 ± 24.15	6.549	0.001
Intraoperative bleeding volume (mL)	197.68 ± 136.15	340.00 ± 150.54	3.910	< 0.001

### Radiographic findings

The PHR, the Cobb angle, and the MSDCR in the two groups after surgery and at the last follow-up were significantly improved when compared with the preoperative values (all *P* < 0.05, *Table **3*). No significant difference in PHR and Cobb angle was found between the two groups after the operation and at the last follow-up (*P* > 0.05), but improvements in MSDCR were observed at the last follow-up compared with the postoperative values (*P* < 0.05).

**Table 3:** Results for within-group and between-group comparisons at each time point

Variables	Time	Group A (n = 28)	Group B (n = 36)	*t* values	*P* values
PHR (%)	Pre-operation	60.77 ± 9.75	46.65 ± 11.91	5.085	< 0.001
	Post-operation	97.79 ± 3.27*	97.56 ± 7.29*	0.157	0.875
	Last follow-up	96.84 ± 3.49*	96.83 ± 7.62*	0.004	0.997
	*F* values	183.492	375.564		
	*P* values	< 0.001	< 0.001		
Cobb angle (°)	Pre-operation	9.71 ± 5.08	14.94 ± 5.72	3.811	< 0.001
	Post-operation	5.32 ± 2.16*	5.89 ± 3.23*	0.800	0.427
	Last follow-up	4.96 ± 2.22*	6.44 ± 3.35*	2.017	0.048
	*F* values	17.592	43.882		
	*P* values	< 0.001	< 0.001		
MSDCR (%)	Pre-operation	55.91 ± 6.70	56.21 ± 7.10	0.167	0.868
	Post-operation	10.11 ± 4.99*	8.34 ± 2.77*	1.799	0.077
	Last follow-up	8.15 ± 4.83*^#^	6.22 ± 2.54*^#^	2.061	0.044
	*F* values	499.306	907.014		
	*P* values	< 0.001	< 0.001		

*PHR* prevertebral height ratio, *MSDCR* mid-sagittal canal diameter compression ratio, *Group A* MOT group, *Group B* traditional open surgery group

*P < 0.05 compared with preoperative

^#^*P* < 0.05 compared with postoperative

The preoperative PHR was heavier in group B than in group A (*P* < 0.05), but there was no significant difference between the postoperative and last follow-up values (*P* > 0.05). The Cobb angle in group B was larger than in group A before the operation (*P* < 0.05), showing no significant difference from the postoperative values (*P* > 0.05). However, the Cobb angle in group A was better maintained than in group B at the last follow-up (*P* < 0.05). No significant difference in MSDCR was observed in the two groups between the preoperative and post-operation values (*P* > 0.05). In contrast, the MSDCR in group B showed a greater improvement than in group A at the last follow-up (*P* < 0.05).

### Visual analogue scale (VAS) score

There was no significant difference in VAS score between the groups before operation (*P* > 0.05, *Table **4*). The VAS score on postoperative day 1 and at the last follow-up was significantly lower than the respective preoperative values in both groups (*P* < 0.05). However, the improvement in VAS score was significantly more favorable in Group A than in Group B (*P* < 0.05).

**Table 4:** Comparison of VAS score

Time	Group A (n = 28)	Group B (n = 36)	*t* values	*P* values
Pre-operation	7.04 ± 0.92	6.97 ± 0.97	0.976	0.333
postoperative day 1	2.89 ± 0.69*	3.39 ± 0.87*	2.475	0.016
Last follow-up	0.36 ± 0.49*^#^	0.67 ± 0.54*^#^	2.386	0.020
*F* values	577.701	775.714		
*P* values	< 0.001	< 0.001		

*P < 0.05 compared with preoperative

^#^*P* < 0.05 compared with postoperative day 1

### American Spinal Injury Association (ASIA) scores

The ASIA scores demonstrated significant differences between the preoperative values and the last follow-up values in the two groups (*P* < 0.05, *Table **5*).

**Table 5:** Comparison of ASIA scores

ASIA score	Group A		Group B	
	Pre-operation	Last follow-up	Pre-operation	Last follow-up
A	0	0	8	2
B	0	0	6	3
C	12	1	5	6
D	13	14	11	11
E	3	13	6	14
*Z* value	3.860		2.593	
*P* value	< 0.001		0.010	

The rank order of ASIA scores in this article were (A)-1, (B)-2, (C)-3, (D)-4, and (E)-5; Group A: preoperative mean rank nontagged 20.73 and the last follow-up mean rank nontagged 36.27; Group B: preoperative mean rank nontagged 30.29 and the last follow-up mean rank nontagged 42.71

### A typical case of MOT

**Fig. 3:**
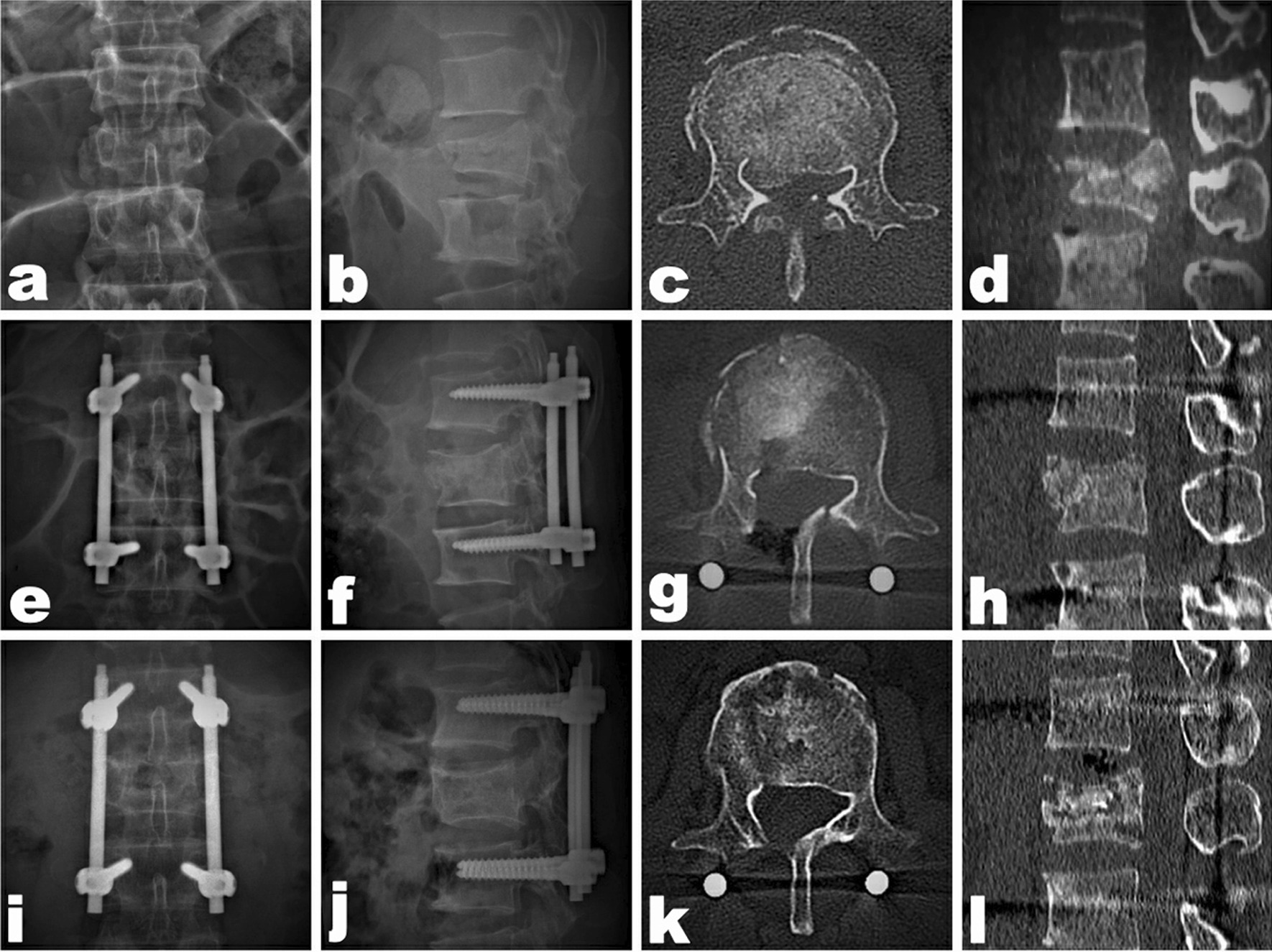
A 47-year-old man with L2 burst fracture (AO type A4) with severe traumatic spinal stenosis. **a** Preoperative anteroposterior radiograph; **b** preoperative lateral radiograph; **c** preoperative CT axial view; **d** preoperative CT sagittal view; **e** postoperative anteroposterior radiograph; **f** postoperative lateral radiograph; **g** postoperative CT axial view; **h** postoperative CT sagittal view; **i** 2-year postoperative anteroposterior radiograph; **j** 2-year postoperative lateral radiograph; **k** 2-year postoperative CT axial view; and **l** 2-year postoperative CT sagittal view

A 47-year-old male patient was diagnosed with AO type A4 fractures at the L2 level with a large posterior wall retropulsed fragment into the spinal canal, causing significant spinal canal encroachment, exhibiting ASIA E neurological status. MOT treatment resulted in improvements in the prevertebral height and Cobb angle, and the fragment in the spinal canal showed a good reduction. At the last follow-up, the patient had ASIA E neurological status, and the radiographic results showed good fracture union. Although partial bone graft resorption was observed, the prevertebral height, Cobb angle, and spinal canal patency were well maintained, see Fig. 3.

## Discussion

### The definition of severe traumatic spinal stenosis caused by thoracolumbar burst fractures

The definition of severe traumatic spinal stenosis of thoracolumbar fractures remains controversial. Wolter [12] defined a retropulsed fragment into the spinal canal causing MSDCR > 2/3 as severe spinal stenosis. Meves et al. [13] reported a positive correlation between spinal canal narrowing and the severity of the incomplete neurological deficit. Patients with 25, 50, and 75% narrowing of the thoracolumbar spinal canal exhibited a 12, 41, and 78% probability of neurological deficit, respectively. In the lumbar spinal canal, the probability was 8, 30, and 68%, respectively. Based on our previous experience, patients with MSDCR > 50% have a high probability of neurological deficit, which coincided with the findings of previous studies by Mohanty et al. [14] Therefore, MSDCR > 50% is recommended as a standard to define severe traumatic spinal stenosis due to difficult fracture reduction and the high probability of neurological deficit.

### Advantages of MOT

MOT combines the advantages of minimally invasive procedures and open surgeries: (a) Percutaneous pedicle screw fixation and fenestration of the vertebral lamina were performed via the minimally invasive incisions while protecting the paraspinal muscle and PLC. Thoracolumbar segmental stability was maintained, and postoperative pain and bleeding were reduced [4]; (b) the fracture fragments can be directly and indirectly pressed into the fractured vertebral body using the L-shaped operative tool and the elastic tension of the posterior longitudinal ligament; and c) satisfactory anterior and middle column reduction and adequate bone graft can be achieved via the spinal canal (Fig. 3). In these studies, the postoperative PHR, Cobb angles, and MSDCR were improved compared with preoperative values (*P* < 0.05). The findings suggested that both the MOT and traditional open surgery effectively achieve decompression of the spinal canal, correct spinal deformity, and rebuild spinal stability. No significant difference was found in the PHR and the Cobb angle in the two groups between the postoperative values and the last follow-up values (*P* > 0.05), but the Cobb angle in group A was better maintained than in group B at the last follow-up (*P* < 0.05). Although both procedures can restore and maintain spinal stability, the MOT yields better results than traditional open surgery. The VAS score of the two groups was significantly lower after surgery (*P* < 0.05), while the VAS score of group A was lower than group B on postoperative day 1 and at the last follow-up (*P* < 0.05). MOT resulted in excellent pain relief. Four pedicle screw techniques are still controversial from the biomechanical point of view. However, no internal fixation failure was found during follow-up in this study. Considering the absence of serious PLC injury and dislocation in type A3 and A4 fractures, through adequate bone grafting in the vertebral body, correction of kyphosis, rigid fixation with four pedicle screws, protection of paraspinal muscles, and external fixation of the thoracolumbar brace, we believe that four pedicle screw techniques are effective for type A3 and A4 fractures.

### The significance of using a surgical microscope in MOT

Due to the small and deep incision for decompression, a restricted view of the surgical area and inadequate lighting, such as in traditional open surgery, hinders the decompression procedure and compromises safety. The surgical microscope can compensate for the above limitations. The major advantages of the microscope include better illumination, magnification, and coaxial vision, which contribute to avoiding spinal cord and nerve root injuries as well as dural lesions [15]. The intraoperative bleeding volume of MOT was less than traditional open surgeries (*P* < 0.05). One major reason is the application of the surgical microscope, as the perivertebral venous plexus anatomy can be identified intraoperatively and controlled with compression hemostasis and electrocautery accurately [16].

### Experience in MOT

#### Details of decompression and reduction

Type A3 fractures are incomplete burst fractures affecting a single endplate and the posterior wall. Type A4 fractures are complete burst fractures involving both endplates, with the involvement of the posterior cortex, a greater degree of height loss, and possible spinal canal encroachment. The latter is responsible for the highest incidence of neurological injury of all A subtypes. Depending on the severity of compression of the nerve root and spinal cord, different orders of decompression and reduction should be considered [17]. When neural tissue is severely compressed or immobilized by bony fragments in the spinal canal or fractured lamina, especially at the T11-12 vertebral level, the decompression and reduction should be performed in the following order: fenestration → longitudinal distraction → reduction by curette. Type A4 fractures are usually treated in this order. In the absence of serious compression or incarceration of neural tissue, the decompression and reduction should be performed as follows: longitudinal distraction → fenestration → reduction by curette. Type A3 fractures are usually treated in this order. Fenestration should be performed on the side of the neural tissue compression or severe injury. Unilateral fenestration is recommended unless the bilateral lamina or lateral wall of the spinal canal was fractured seriously.

### Bone graft skills

In our previous study, sagittal fracture lines were observed over the pedicle horizontal in thoracolumbar fractures with loss of vertebral body height. This region overlaps with the interlaminar space, so a 3-cm incision at this region is sufficient for decompression, reduction, and bone graft. As the adjacent disks and PLC were not severely damaged, the MOT did not require posterolateral fusion or interbody fusion. Fixation was performed by the posterior approach and bone graft did not induce significant changes in the intervertebral spaces, and the anterior column achieved spontaneous fusion [6, 18–20]. In this study, the postoperative PHR and Cobb angle improved significantly (*P* < 0.05) and remained stable at the last follow-up (*P* < 0.05). These results are in accordance with the above points.

### Treatment strategy for the spinal canal

Anatomic reduction is not necessary if the process may cause damage to the spinal cord or nerves. Miyashita et al. [21] found that there is no significant effect on the recovery of neurological function when MSDCR < 30%. After removing the fragments or soft tissues compressing the neural structures, bone resorption can complete spinal canal remodeling. This is related to intraspinal venous pulsation [22]. The results of this study showed no significant difference in MSDCR between preoperative and postoperative values in the two groups (*P* > 0.05), revealing that both the MOT and traditional open surgery effectively achieve decompression of the spinal canal. However, the MSDCR in traditional open surgery at the last follow-up (6.22 ± 2.54%) was superior to MOT (8.15 ± 4.83%) (*P* < 0.05). The mechanisms involved remain unclear and require further investigation.

### Limitations in this study

This was a retrospective study with small sample size and a short follow-up time. To confirm the long-term effectiveness and advantages of MOT, the sample size should be expanded in further research. Meanwhile, the effectiveness after removing the internal fixation requires further investigation.

## Conclusions

Both the MOT and traditional open surgery effectively treat AO type A3 and A4 thoracolumbar burst fractures with severe traumatic spinal stenosis. The advantages of MOT include minimal invasion, extremely fine spinal canal decompression, lower intraoperative bleeding, and obvious pain relief. We suggest that MOT should be preferentially performed for AO type A3 or A4 thoracolumbar burst fractures with severe traumatic spinal stenosis.

## Data Availability

The datasets used and/or analyzed during the current study are available from the corresponding author on reasonable request.

## Abbreviations

MOT: Minimal invasive decompression by posterior microscopic mini-open technique combined with percutaneous pedicle fixation
AO: Arbeitsgemeinschaft für Osteosynthesefragen classification
ASIA: American Spinal Injury Association score
VAS: Visual analogue scale score
PHR: Prevertebral height ratio
MSDCR: Mid-sagittal canal diameter compression ratio
PLC: Posterior ligamentous complex
